# Partial depletion of dopaminergic neurons in the substantia nigra impairs olfaction and alters neural activity in the olfactory bulb

**DOI:** 10.1038/s41598-018-36538-2

**Published:** 2019-01-22

**Authors:** Wenfeng Zhang, Changcheng Sun, Yufeng Shao, Zheng Zhou, Yiping Hou, Anan Li

**Affiliations:** 1 Jiangsu Key Laboratory of Brain Disease and Bioinformation, Research Center for Biochemistry and Molecular Biology, Xuzhou Medical University, Xuzhou, China; 2 Department of Neuroscience, Anatomy, Histology, and Embryology, Key Laboratory of Preclinical Study for New Drugs of Gansu Province, School of Basic Medical Sciences, Lanzhou University, Lanzhou, China

## Abstract

Olfactory dysfunction is a major non-motor symptom that appears during the early stages of Parkinson’s Disease (PD), a neurodegenerative disorder characterized by loss of dopaminergic neurons in the substantia nigra (SN). Depletion of SN dopaminergic neurons by 6-hydroxydopamine (6-OHDA) is widely used as a model for PD and ultimately results in motor deficits. However, it is largely unknown whether olfactory behavior and, more importantly, neural activity in the olfactory bulb (OB) are impaired prior to the appearance of motor deficits. We partially depleted the SN dopaminergic population in mice by injection of 6-OHDA. Seven days after injection of 6-OHDA, motor ability was unchanged but olfactory-driven behaviors were significantly impaired. Injection of 6-OHDA into the SN significantly increased the power of the ongoing local field potential in the OB for all frequency bands, and decreased odor-evoked excitatory beta responses and inhibitory high-gamma responses. Moreover, 6-OHDA treatment led to increased odor-evoked calcium responses in the mitral cells in the OB of awake mice. These data suggest that the olfactory deficits caused by depletion of the SN dopaminergic population are likely due to abnormal hyperactivity of the mitral cells in the OB.

## Introduction

Parkinson’s disease (PD) is a neurodegenerative disorder characterized by massive loss of dopaminergic neurons in the substantia nigra (SN) and subsequent dopamine depletion in the striatum, a critical structure for motor control. These changes induce progressive, irreversible, and ultimately disabling motor deficits^1–3^. Besides motor dysfunction, many non-motor symptoms are reported with PD, such as hyposmia, dysphagia, anxiety, and constipation. Among these, smell dysfunction is the most salient non-motor feature of PD and occurs in at least 90% of cases, often years prior to the motor disturbances^4–7^. Although it is widely accepted that hyposmia occurs during the early stages of PD and may be a possible biomarker of PD^5,8^, little is known about the underlying neural mechanisms.

The olfactory bulb (OB) is the first processing hub in the olfactory system, and is important for the representation of odor identity, intensity, and timing^9–15^. While the dopaminergic neurons in the SN play an important role in PD, the OB also contains a population of dopaminergic interneurons. These are primarily located within the glomerular layer (GL)^16,17^, where they comprise approximately 10% of GL neurons. In the GL, olfactory sensory neurons synapse with mitral/tufted cells (M/T cells), which are the main output neurons of the OB. Both *in vivo* and *in vitro* studies have revealed that the dopaminergic neurons in the OB modulate odor information processing^18–22^ and behavioral odor discrimination^23,24^. Most importantly, a recent study has demonstrated the existence of a direct axonal dopaminergic projection from the SN to the external plexiform layer (EPL), granule cell layer (GCL), and mitral cell layer (MCL) of the OB, and ablation of this projection impaired olfactory perception^1^. Therefore, it is likely that olfactory dysfunction during the early stages of PD is due to abnormal dopaminergic modulation of the OB.

In the OB, odor information is processed and represented by variety of different types of neurons and related neural circuits^25^. M/T cells are the major output neurons so their activity reflects the end result of neural information processing in the OB^14,25^. At the network scale, the beta and gamma oscillations in the local field potential (LFP) recorded from the OB are also critically involved in odor discrimination and olfactory learning^26,27^. It is surprising that although a large number of previous studies have investigated the pathological features of the OB in PD mouse models, no research has assessed how the neural activity of the OB changes during the very early stages of PD.

To address this, and to investigate whether dopaminergic neurons in the SN affect neural activity in the OB and olfaction-related behaviors in mice, we used the dopaminergic neurotoxin 6-hydroxydopamine (6-OHDA) to partially deplete the SN dopaminergic population as a model of the early stages of PD. On the seventh day after 6-OHDA injection, the motor ability of the mice was unchanged but there was neural hyperactivity in the OB and olfactory-driven behaviors were significantly impaired.

## Results

### Partial depletion of dopaminergic neurons in the SN seven days after injection of 6-OHDA

**Figure 1:**
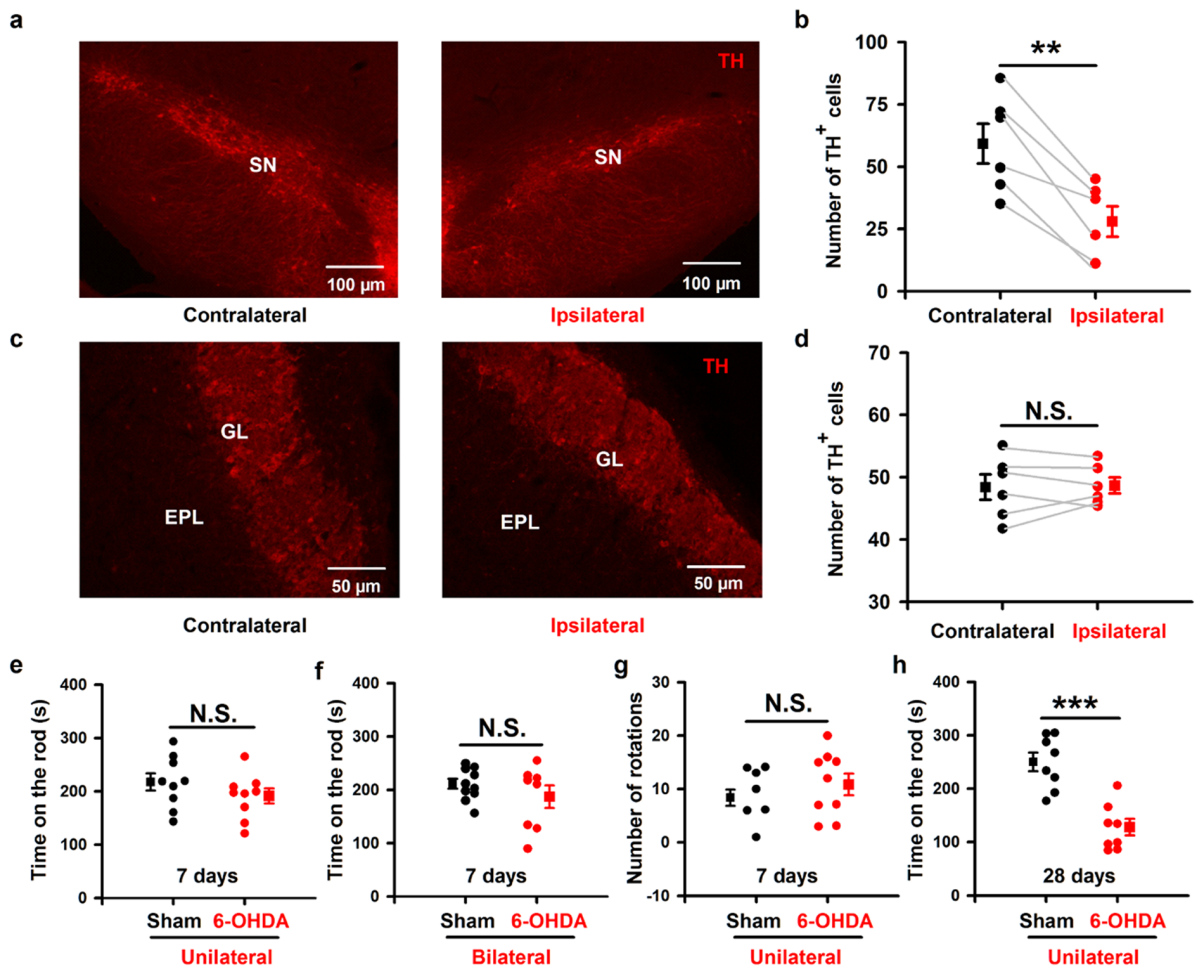
No significant locomotor impairments in mice with partial depletion of SN dopaminergic neurons after injection of 6-OHDA. (**a**,**c**) TH immunostaining in SN (**a**) and OB (**c**) from a representative mouse. Left and right images are contralateral and ipsilateral to the 6-OHDA SN injection site, respectively. (**b**,**d**) Comparison of the number of TH^+^ cells in the ipsilateral and contralateral SN (**b**) and OB (**d**) after unilateral SN 6-OHDA across all the 6 mice. (**e**,**f**) Time on the rod in the rotarod test for 6-OHDA treated mice and sham mice injected unilaterally (**e**) or bilaterally (**f**). (**g**) Number of apomorphine-induced rotations in 30 minutes in 6-OHDA treated mice and sham mice. (**h**) Time on the rod in the rotarod test in 6-OHDA treated mice and sham mice 28 days after drug injection. Error bars represent s.e. **P < 0.01; ***P < 0.001; N.S., not significant.

To verify the ability of 6-OHDA to lesion dopaminergic neurons in the SN, we injected 2 µg 6-OHDA into the SN unilaterally (see Methods for details). Seven days after the stereotaxic injection, immunohistochemical analysis showed that there were fewer tyrosine hydroxylase positive (TH^+^) cells in the SN on the 6-OHDA-injected side (ipsilateral) than in the contralateral SN (Fig. 1a). Overall, the ipsilateral SN contained approximately half as many dopaminergic neurons as the contralateral SN (Fig. 1b, 59.1 ± 8.0 cells vs. 27.8 ± 6.1 cells, paired *t*-test, *P* = 0.002, *n* = 6 mice). Thus, our procedure was effective in lesioning dopaminergic neurons in the SN.

Since the OB glomerular layer also contains a substantial population of dopaminergic neurons, application of 6-OHDA into the SN might lesion these dopaminergic neurons in the OB in addition to the dopaminergic neurons in the SN. To confirm that dopaminergic neurons were selectively depleted only in the SN and not in the OB, we counted the number of dopaminergic neurons in the OB glomerular layer seven days after injection of 6-OHDA into the SN (Fig. 1c). The number of TH^+^ immunolabeled cells (mainly periglomeruli cells, PGCs) in the ipsilateral and contralateral OB were not significantly different (Fig. 1d, 48.6 ± 1.3 cells vs. 48.3 ± 2.0 cells, paired *t*-test, *P* = 0.82, *n* = 6 mice). This is consistent with a recent study that performed a similar lesion experiment in rats^28^. Therefore, 6-OHDA treated mice are an animal model of early-stage PD (because of the significant decrease in SN dopaminergic neurons) but are not a model of lesion of OB interneurons.

### Partial depletion of SN dopaminergic neurons does not affect motor ability

Next, we checked whether 6-OHDA mice had impaired locomotor skills. We tested sham mice (injected unilaterally with 0.9% saline containing 0.02% ascorbic acid) and 6-OHDA mice on a rotarod seven days after injection. The falling latencies (time on the rod) were not significantly different for the 6-OHDA treated mice and the sham mice (Fig. 1e, 216.6 ± 16.3 s and 190.2 ± 14.1 s for sham and 6-OHDA treated mice, respectively; unpaired *t*-test, *P* = 0.24, *n* = 9 mice in each group). To further confirm this finding, we injected 6-OHDA into the SN bilaterally in a separate experiment. We again found that the falling latencies were not significantly different for the bilaterally injected 6-OHDA treated mice and the sham mice (Fig. 1f, 210.0 ± 9.5 s and 185.4 ± 21.0 s for sham and 6-OHDA treated mice, respectively; unpaired *t*-test, *P* = 0.27, *n* = 10 mice (sham) and *n* = 8 mice (6-OHDA)). If the 6-OHDA treated mice are indeed a model of early-stage PD animal model, then these mice would be expected to ultimately display motor deficits. We verified this by testing the motor ability of treated mice four weeks after 6-OHDA injection. In this case, we found a significant difference between the falling latencies for 6-OHDA treated mice and sham mice (Fig. 1h, 244.7 ± 17.7 s and 122.3 ± 15.5 s for sham and 6-OHDA treated mice, respectively; unpaired *t*-test, *P* < 0.001, *n* = 8 mice in each group), indicating a substantial motor deficit four weeks after 6-OHDA injection.

Motor ability was also evaluated by the apomorphine-induced rotation test seven days after 6-OHDA injection. We found no significant difference between the sham mice and the 6-OHDA mice in the number of turns over 30 min (Fig. 1g, 8.4 ± 1.5 turns and 10.9 ± 2.0 turns for sham and 6-OHDA mice, respectively; unpaired *t*-test, *P* = 0.35, *n* = 9 mice in each group). These data indicate that 6-OHDA lesion in the SN using our procedure had no functional impact on motor coordination seven days after injection. These findings are highly consistent with previous reports that motor deficits only occur after 6-OHDA injection when more than 90% of the SN dopaminergic neurons have been ablated^29–32^.

### Olfactory-related behaviors are impaired seven days after injection of 6-OHDA

**Figure 2:**
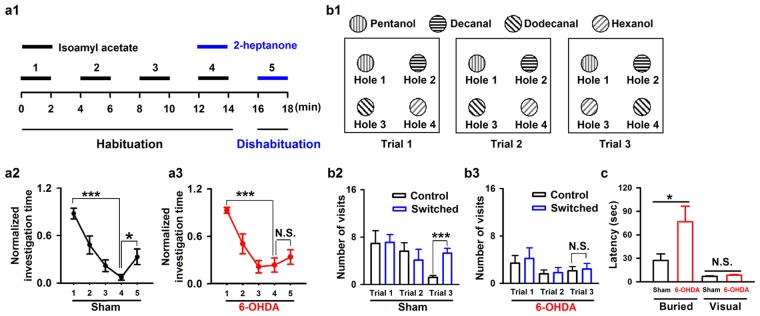
Injection of 6-OHDA into the SN impairs odor discrimination and odor spatial memory in mice. (**a1**) Schematic for the odor habituation/dishabituation test in C57BL/6 J mice. (**a2**,**a3**) Odor habituation (the same odor stimulation repeated four times with 2 min interval, 1^st^–4^th^ trial) and dishabituation (novel odorant on the 5^th^ trial) in sham mice (**a2**, ***n*** = **14**) and 6-OHDA treated mice (**a3**, ***n*** = **14**). (**b1**) Schematic for the olfactory spatial memory test in C57BL/6 J mice. (**b2**,**b3**) Number of visits to the control and switched odors in the training trials (trial 1 and 2) and the recall trial (trial 3) in sham mice (**b2**, ***n*** = **10**) and 6-OHDA treated mice (**b3**, ***n*** = **10**). (**c**) Latency to locate a food pellet in sham and 6-OHDA treated mice for a buried food pellet (Buried) or food on the surface of the bedding (Visual). *P < 0.05; ***P < 0.001; N.S., not significant.

Although the partial depletion of dopaminergic neurons in the SN did not impair motor ability in the mice, it could affect olfactory-driven behaviors since olfactory dysfunction usually appears prior to motor disturbances in PD^4,5,8^. We assessed olfactory function with the habituation/dishabituation test seven days after bilateral 6-OHDA injection into the SN in mice. Performance during odor habituation/dishabituation reflects olfactory discrimination and memory, and this spontaneous odor discrimination task has been widely used in previous studies^24,33,34^. Mice were presented with the same odorant four times, after which a new odorant was presented (Fig. 2a1, see Methods for details). Successful dis-habituation between the fourth repeated odor presentation and the novel odor presentation indicate the ability to discriminate odors, and habituation across the four repeated odor presentations is linked to odor memory. For sham mice, the time spent investigating the odor decreased with repeated odor stimulation (Fig. 2a2, one-way ANOVA, *F*_*(4,65*_) = 10.14, *P* < 0.001; 1^st^ vs. 4^th^ trial, *P* < 0.001), and increased significantly when mice were exposed to the novel odorant (Fig. 2a2, 4^st^ vs. 5^th^ trial, *P* < 0.05). For 6-OHDA treated mice, the investigation time also decreased with repeated odor presentation (Fig. 2a3, One-way ANOVA, *F*_*(4,65)*_ = 10.76, *P* < 0.001; 1^st^ vs. 4^th^ trial, *P* < 0.001); however, the investigation time did not increase significantly when mice were exposed to the novel odorant (Fig. 2a3, 4^st^ vs. 5^th^ trial, *P* = 0.42). These results suggest that our SN 6-OHDA treatment impaired the ability of mice to discriminate odors.

We also tested olfactory spatial memory in the 6-OHDA treated mice^35,36^. There was no difference in the number of visits by sham mice to the control and switched odorants during the training trials (trial 1 and 2). In the recall trial (trial 3) the spatial location of two of the odorants was switched (Fig. 2b1). In the recall trial, sham mice visited the switched odorants significantly more often than the control (unswitched) odorants (Fig. 2b2, 1.2 ± 0.3 visits vs. 5.3 ± 0.8 visits, unpaired *t*-test, *P* < 0.001, *n* = 10 mice). However, the 6-OHDA mice made approximately the same number of visits to the switched odorants and the control odorants in the recall trial (Fig. 2b3, 2.1 ± 0.7 visits vs. 2.4 ± 1.0 visits, unpaired *t*-test, *P* = 0.81, *n* = 10 mice). These results indicate that our SN 6-OHDA injection impaired olfactory spatial memory in mice.

To further confirm that the olfactory ability of 6-OHDA treated mice was impaired, we tested the mice with a buried pellet test. In this experiment, we tested the latency to locate a pellet of food buried beneath the bedding or placed on the surface of the bedding (visual condition). The 6-OHDA treated mice took significantly longer to locate the buried pellet than the sham mice (Fig. 2c, 27.2 ± 8.5 s vs. 76.6 ± 19.9 s, unpaired *t*-test, *P* = 0.026, *n* = 10 mice (sham) and *n* = 8 mice (6-OHDA)). However, the sham and 6-OHDA treated mice took a similar length of time to locate the food on the surface of the bedding (Fig. 2c, 6.8 ± 0.7 s vs. 8.4 ± 0.9 s, unpaired *t*-test, *P* = 0.16, *n* = 10 mice (sham) and *n* = 8 mice (6-OHDA)). These results indicate that although olfactory ability is impaired in 6-OHDA treated mice, their motivation, mobility, and attention remain unchanged. Taken together, these three tests of olfactory-related behaviors suggest that loss of about 50% of the dopaminergic neurons in the SN results in olfactory dysfunction but no significant motor deficits.

### Lesion of SN dopaminergic neurons increases the spectral power of the spontaneous LFP recorded in the dorsolateral region of the OB but decreases the odor-evoked LFP responses

**Figure 3:**
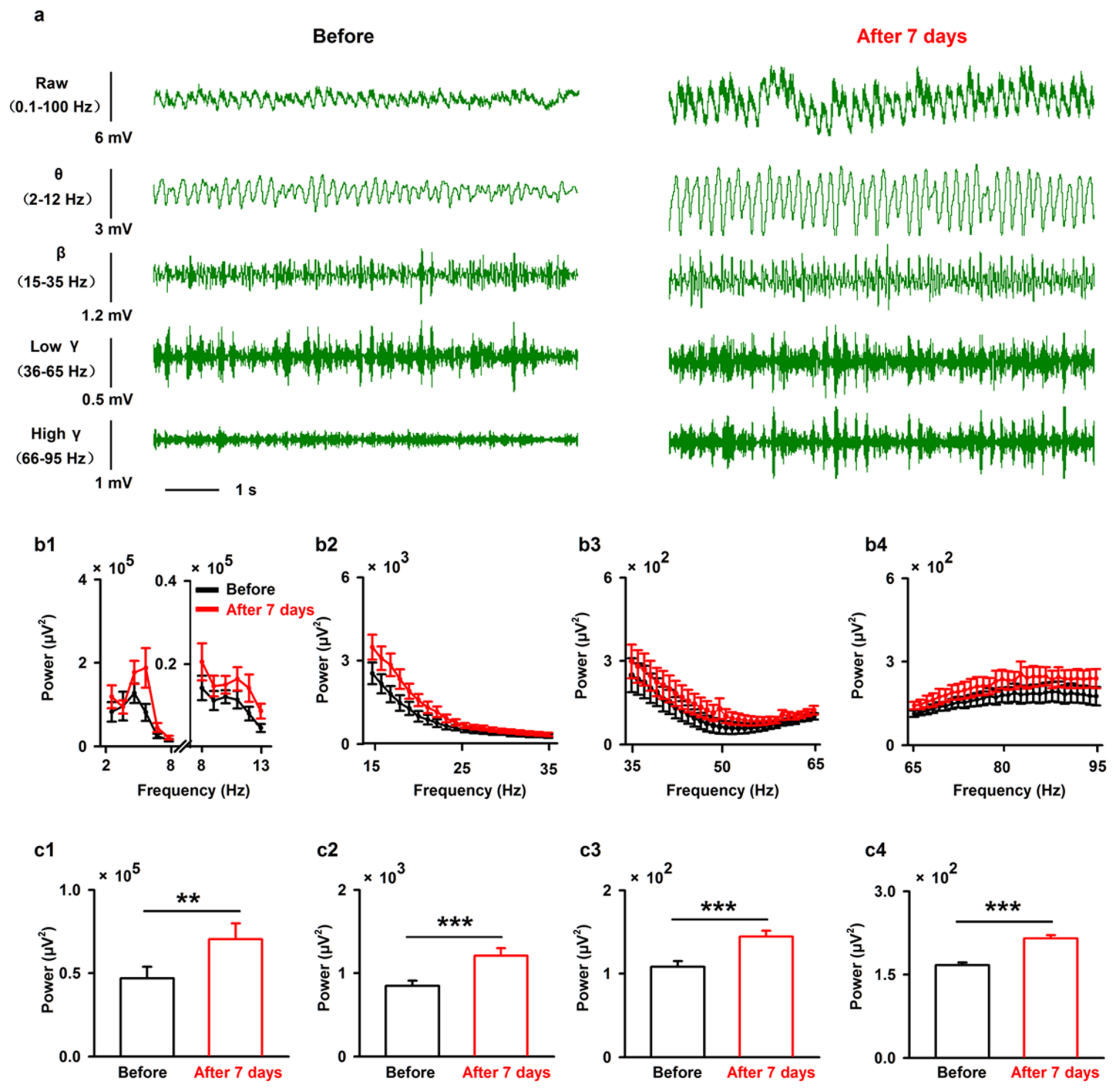
Injection of 6-OHDA increases the power of spontaneous LFP oscillations in the OB in all frequency bands. (**a**) Sample ongoing LFP signals from one mouse before (left) and seven days after (right) injection of 6-OHDA. The first row shows the raw trace (8 s), the second to the fifth rows show filtered signals: θ (2 to 12 Hz), β (15 to 35 Hz), low-gamma (36 to 65 Hz), and high-gamma (66 to 95 Hz). (**b**) Averaged power spectra of theta (b1), beta (b2), low-gamma (b3), and high-gamma (b4) bands across the group of mice recorded. (**c**) Statistical analysis of the power in the different bands (theta (c1), beta (c2), low-gamma (c3), and high-gamma (c4)) before and seven days after 6-OHDA injection. A two-way ANOVA (with 6-OHDA/sham and frequency as the two factors) was performed to test the significance of the effect of 6-OHDA treatment on the LFP. Error bars represent s.e. **P < 0.01; ***P < 0.001.

Next, we investigated the neural basis for olfactory dysfunction in the 6-OHDA treated mice through *in vivo* electrophysiological recordings in the OB, the first relay station in the olfactory system. We compared the LFP signals in awake, head-fixed mice before and seven days after 6-OHDA injection into the SN. As in previous studies^11,37^, we divided the raw LFP signals into different frequency bands: theta, 2–12 Hz; beta, 15–35 Hz; low-gamma, 36–65 Hz; and high-gamma, 66–95 Hz (Fig. 3a). As shown in the representative traces in Fig. 3a, the power of the ongoing spontaneous LFP increased in all four frequency bands seven days after 6-OHDA injection. This effect was maintained when we analyzed the LFPs from all the mice recorded (Fig. 3b,c, two-way ANOVA, *n* = 8 mice, theta: *F*_*(1,140)*_ = 7.88, *P* = 0.006; beta: *F*_*(1,280)*_ = 32.60, *P* < 0.001; low-gamma: *F*_*(1,420)*_ = 17.57, *P* < 0.001; high-gamma: *F*_*(1,420)*_ = 40.79, *P* < 0.001). Furthermore, since the theta oscillation band used in the present study (2–12 Hz) is sometimes broken down into 2–8 Hz and 8–13 Hz bands^38^, we also analyzed these two frequency bands separately. Similar to the results for 2–12 Hz, injection of 6-OHDA significantly increased the power in both the 2–8 Hz and 8–13 Hz frequency bands of the ongoing LFP across all the mice recorded (*F*_*(1,84)*_ = 6.79, *P* = 0.011 for 2–8 Hz; *F*_*(1,84)*_ = 10.07, *P* = 0.002 for 8–13 Hz).

**Figure 4:**
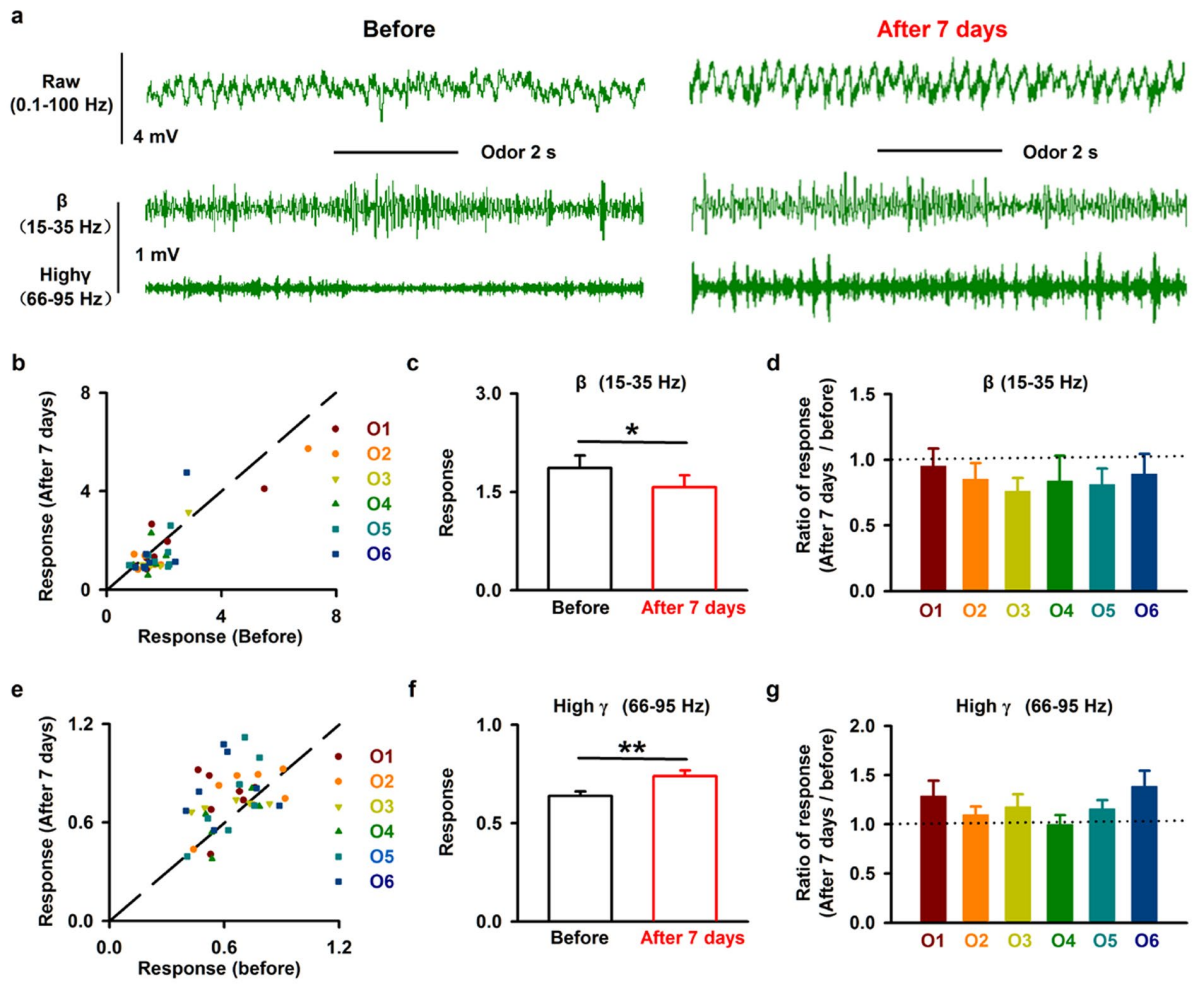
Injection of 6-OHDA decreases the odor-evoked LFP response in the OB. (**a**) Raw LFP traces (top row), filtered beta band (middle row), and filtered high-gamma bands (bottom row) in response to odor (isoamyl acetate) stimulation from one mouse before (left) and seven days after (right) 6-OHDA application. (**b**,**e**) Normalized odor-evoked beta (**b**) and high-gamma (**e**) responses before and seven days after 6-OHDA injection for all animals and odors (*n* = 38). The dotted line shows the diagonal, where the response amplitude before 6-OHDA injection is equal to that seven days after 6-OHDA application. O1–O6 indicate isoamyl acetate, 2-heptanone, phenyl acetate, benzaldehyde, dimethylbutyric acid, and n-Heptane acid, respectively. (**c**,**f**) Comparison of odor-evoked normalized beta responses (**c**) and high-gamma responses (**f**) before and seven days after 6-OHDA injection across the group of mice recorded. *P < 0.05; **P < 0.01. (**d**,**g**) Normalized change in the odor-evoked beta (**d**) and high-gamma (**g**) responses (seven days after/before) to six odorants; the dashed line indicates 1, where the response before 6-OHDA application is equal to the response seven days after 6-OHDA treatment.

We also investigated the effects of 6-OHDA injection on odor-evoked LFP responses in awake mice. Figure 4a shows an example of the LFP response to isoamyl acetate in an awake mouse, with a strong excitatory response in the beta band and an inhibitory response in the high-gamma band, similar to our previous recordings in awake head-fixed mice^37^. Seven days after injection of 6-OHDA into the SN, the isoamyl-acetate-induced excitatory beta band response and inhibitory high-gamma band response were both reduced (Fig. 4a). This phenomenon was also observed for other odorants (Fig. 4b,e).

When we measured the response across different mice and odors, we found that injection of 6-OHDA into the SN significantly reduced both the odor-evoked excitatory beta response (Fig. 4c, paired *t*-test, *P* = 0.011, n = 38) and the odor-evoked inhibitory high-gamma response (Fig. 4f, paired *t*-test, *P* = 0.0013, n = 38). Further analysis revealed that the effect did not differ significantly among odors for either the beta or high-gamma responses (Fig. 4d,g, *F*_*(5,32)*_ = 0.22 and *F*_*(5,32)*_ = 1.16, *P* = 0.95 and *P* = 0.35 for beta and high-gamma, respectively, one-way ANOVA). Therefore, these data indicate that partial lesion of the SN dopaminergic neurons reduces the extent of odor-evoked responses in the OB.

### Lesion of SN dopaminergic neurons increases odor-evoked calcium responses in the mitral cells

**Figure 5:**
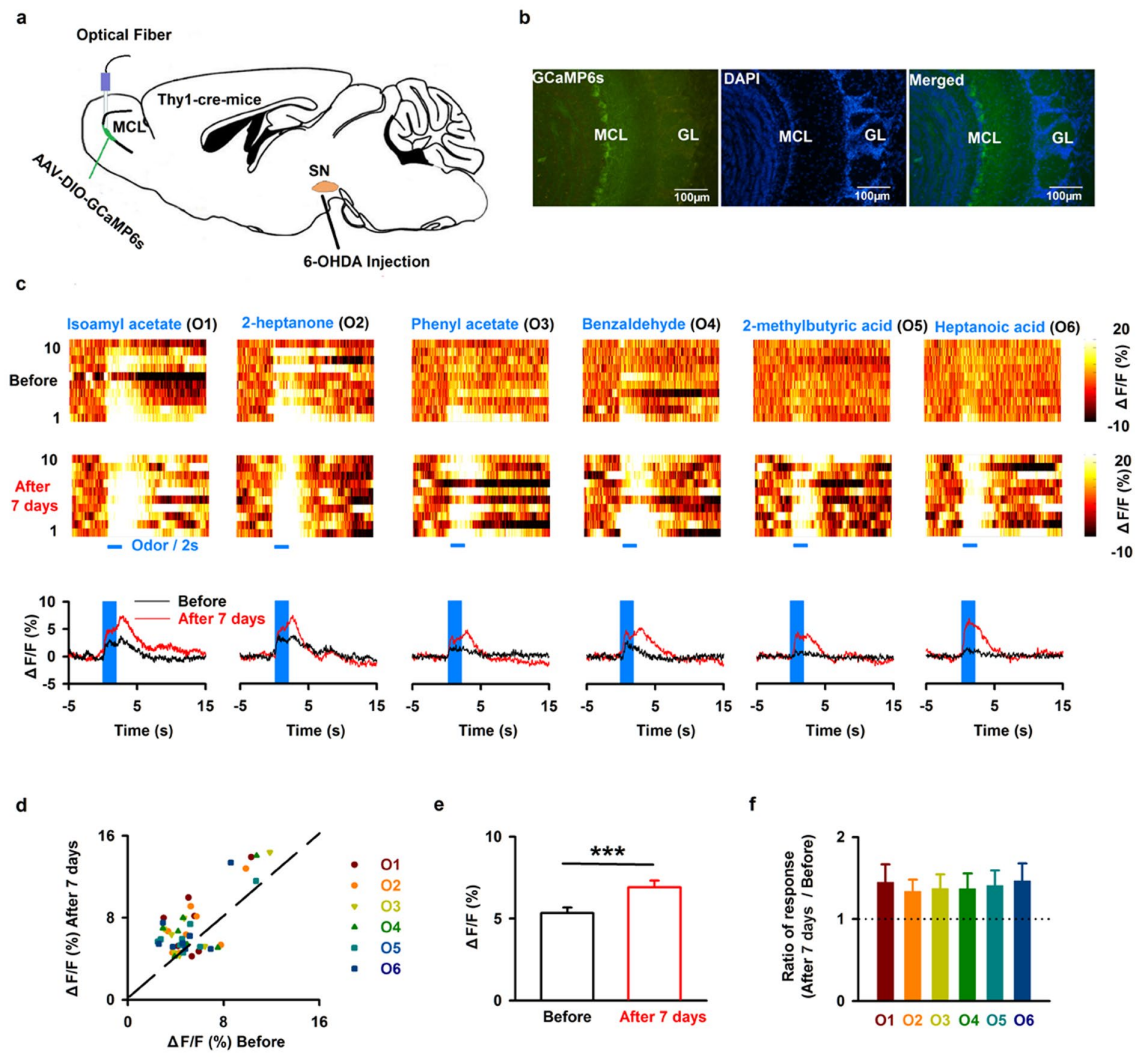
Injection of 6-OHDA enhances odor-evoked mitral cell calcium responses in the OB. (**a**) Schematic for recording calcium signals by fiber photometry from mitral cells expressing GCaMP6s in Thy1-cre mice. (**b**) Expression of GCaMP6s in the MCL. (**c**) Representative calcium transient hit maps in response to six different odors from one mouse before (upper row) and seven days after 6-OHDA injection (middle row). The plots (bottom row) show the average odor-evoked calcium response from ten trials before (black line) and seven days after 6-OHDA application (red line). (**d**) Normalized odor-evoked calcium responses before 6-OHDA injection and seven days after 6-OHDA injection for all animals and odors (*n* = 48, 8 mice with 6 odors). The dotted line shows the diagonal, where response amplitude before 6-OHDA application is equal to that seven days after 6-OHDA application. (**e**) Odor-evoked calcium responses before and seven days after 6-OHDA treatment across the group of mice recorded. Error bars represent s.e. ***P < 0.001 (**f**). Normalized change in the odor-evoked calcium responses (seven days after/before) to six odorants; the dashed line indicates 1, where the response before 6-OHDA injection is equal to the response after 6-OHDA treatment.

The LFP reflects the activity of neural networks in the OB and thus contains important information about basic olfactory processing. Changes in the LFP are related to olfactory cognition and likely also to additional functions such as olfactory-associated rewarding behavior^27^. However, the mitral cells are the main output neurons of the OB, and the activity in these cells is crucial for odor representation and further information decoding in downstream centers such as the piriform cortex^14^. Thus, we investigated whether 6-OHDA injection affects the odor-evoked population activity of mitral cells in awake mice. We used fiber photometry to record calcium signals specifically from mitral cells. We injected the virus AAV-DIO-GCaMP6s into the OB of Thy1-cre mice, and implanted an optical fiber after the viral injection (Fig. 5a). Ten days later, we found extensive expression of GCaMP6s in the mitral cell layer of the OB (Fig. 5b). Odors evoked an increase in GCaMP6s fluorescence in awake head-fixed mice (Fig. 5c, upper row). Seven days after 6-OHDA application, response amplitudes were dramatically altered for many odors and/or mice (Fig. 5c, bottom row). In general, application of 6-OHDA significantly increased odor-evoked calcium responses across the group of mice recorded (Fig. 5d,e, from 5.30 ± 0.33% to 6.90 ± 0.41%; paired *t*-test, *P* < 0.001, *n* = 48, 8 mice with 6 odors). Further analysis with a one-way ANOVA showed that the increased mitral cells calcium response after 6-OHDA did not differ significantly among odors (Fig. 5f, *F*_*(5*_,_*42)*_ = 0.07, *P* = 0.996). These results indicate that odor-evoked calcium responses in the mitral cells are increased after partial depletion of the dopaminergic neurons in the SN.

## Discussion

Our study provides new information on the behavioral and physiological olfactory dysfunction after partial depletion of dopaminergic neurons in the SN by injection of 6-OHDA. We demonstrate that, seven days after injection of 6-OHDA into the SN, only about half of the dopaminergic neurons remain. At this stage, the motor abilities of the mice are unchanged (Fig. 1) but olfactory-driven behaviors are impaired, including odor discrimination and odor spatial memory (Fig. 2). At the neural network level, injection of 6-OHDA into the SN significantly increases the power of the ongoing OB LFP for all frequency bands (Fig. 3), and decreases the extent of odor-evoked excitatory beta responses and inhibitory high-gamma responses (Fig. 4) in the OB. Moreover, at the level of the output cell population, injection of 6-OHDA into the SN increases odor-evoked calcium responses in the mitral cells in the OB of the awake mice (Fig. 5).

Loss of dopaminergic neurons in the SN is the major cause of PD^1,3^. Clinically, olfactory dysfunction usually occurs several years prior to the onset of motor deficits^4,8^. In animals, studies from both genetic and drug-induced PD mouse models have shown impairments in olfactory-related behaviors, and pathological features in olfactory centers, such as the OB^39–42^. This raises the possibility that the olfactory dysfunction present during the early stages of PD is directly linked to the loss of SN dopaminergic neurons, suggesting that these neurons dramatically modulate the function of the olfactory system. Our study provides further evidence to support this hypothesis by directly lesioning SN dopaminergic neurons and demonstrating subsequent changes in olfactory-related behaviors in mice and odor-evoked neural responses of the OB.

Compared with genetic mouse models of PD, fewer studies have tested olfactory behavior in drug-induced mouse models of PD. There were no changes in olfactory behavior in an MPTP-induced mouse model of PD^43^. However, another more recent study by Valle-Leija and Drucker-Colin (2014) has characterized significant deficits in olfactory behavior in a 6-OHDA-induced PD mouse model^42^. This is consistent with our results showing that odor discrimination and odor spatial memory are severely impaired after SN injection of 6-OHDA. However, motor ability was also impaired in this previous study whereas it was unchanged in ours. This is likely due to differences in the injection site: Valle-Leija and Drucker-Colin injected 6-OHDA into the striatum, which is directly involved in motor control^42^, whereas we injected 6-OHDA into the SN, which is indirectly involved in motor control through its innervation of the striatum.

Olfactory centers in the brain are widely modulated by dopaminergic neurons^44,45^. In particular, there are dopaminergic projections from the SN/striatum to piriform cortex and the olfactory tubercle, higher olfactory centers that play crucial roles in odor cognition and odor-related reward^45,46^. Because of this, the olfactory deficits observed in the 6-OHDA-induced PD mouse model have been thought to be due to dysfunction of these higher centers^42^. Importantly, a recent study revealed the existence of a direct axonal dopaminergic projection from the SN to the OB, including the EPL, GCL, and MCL, and ablation of this projection led to impaired olfactory perception^1^. This indicates that olfactory deficits in the 6-OHDA induced PD mouse model may be due to OB dysfunction resulting from destruction of these dopaminergic projections from the SN. Our direct recordings of neural activity in the OB provide new evidence to support this proposal.

The OB also contains a large population of dopaminergic interneurons within the GL^16^. These interneurons modulate the activity of mitral cells, which are the main output neurons of the OB, and play important roles in olfactory processing and odor representation^25^. Mitral cells express both D1 and D2 dopaminergic receptors^23^. Behaviorally, depletion of either D1 or D2 receptors impairs odor discrimination and perception in rodents^23,24^. Optogenetic activation of the dopaminergic neurons in the OB results in long-range suppression of both the spontaneous and odor-evoked firing of the mitral cells^18^, indicating that dopaminergic modulation has an inhibitory effect on the mitral cells. Furthermore, *in vitro* studies have revealed that dopamine receptor activation in the OB causes significant depression of synaptic transmission at the first relay between olfactory sensory neurons and mitral cells^21^. Given the overall inhibitory effect of intrinsic dopaminergic modulation on mitral cells, it is likely that the recently identified dopaminergic input from the SN to the mitral cells is also inhibitory overall. Therefore, it is not surprising that we found hyperactive network activity after lesion of the dopaminergic neurons in the SN, as reflected by increases in the power of the ongoing LFP oscillations and in the amplitude of odor-evoked calcium responses in the mitral cells.

As the major output from the OB, mitral cells are responsible for sending the olfactory information that has been processed by circuits within the OB to higher olfactory centers, including piriform cortex, the anterior olfactory nucleus, and the olfactory tubercle^14,47^. Thus, the increased odor-evoked calcium response in the mitral cell population indicates that loss of SN dopaminergic modulation causes hyperactivity in the mitral cells, which results in abnormal transmission of neural information from the OB to the higher brain centers responsible for further odor discrimination and cognition. The interaction between the OB and piriform cortex is known to be critically involved in odor perception and discrimination^27,33^. The ongoing beta oscillation in the OB, which is considered to reflect the interaction between the OB and piriform cortex^48,49^, increased after injection of 6-OHDA into the SN. This also indicates that the neural communication between the OB and piriform cortex is hyperactive after 6-OHDA injection. However, the power of the odor-evoked beta oscillation decreased, possibly as a result of higher baseline activity. Abnormal, hyperactive neural activity of the mitral cells and the communication between the OB and piriform cortex, will change the neural activity in piriform cortex, which plays an important role in odor cognition and object separation^46^, ultimately leading to impairments in odor discrimination and odor spatial memory. In future studies, it will be interesting to investigate the changes in neural activity in piriform cortex and the olfactory tubercle after partial depletion of SN dopaminergic neurons.

Besides beta oscillations, gamma oscillations were also significantly changed after injection of 6-OHDA into the SN. The gamma oscillation reflects the local interactions between M/T cells and granule cells in the OB^50,51^. Granule cells form reciprocal dendrodendritic synapses with the lateral dendrites of the M/Ts^25^. The M/T cells send excitatory input to the granule cells, and the granule cells send inhibitory input back to the M/Ts via these synapses, and this connection forms the basis of odor-evoked gamma oscillations in the OB^50^. Thus, the power of the gamma oscillations depends on the activity of the mitral and granule cells. When the mitral cells are hyperactive (e.g. after 6-OHDA injection), increased ongoing gamma oscillatory activity and reduced odor-evoked inhibition of gamma activity are expected. Previous work has shown that gamma oscillatory activity is highly correlated with the difficult odor discrimination task^52^, and disruption of the gamma oscillations results in deficits in odor discrimination in rodents and insects^27,51^. It is therefore likely that the deficits in olfactory-driven behaviors observed in the present study are at least partly due to the abnormal activity of the high-gamma oscillation.

The dorsal and ventral zones of the OB play different roles in odor discrimination and detection^53^, and a recent study revealed that the olfactory receptors linked to the dorsal OB are critically involved in enantiomer identification^54^. In the present study, we recorded LFPs and calcium transients from the dorsolateral part of the OB. It is possible that only the dorsal part of the OB is affected by injection of 6-OHDA into the SN. However, direct evidence for this would need to be provided in future studies.

Although we propose that the altered neural activity in the OB is the result of damage to a direct dopaminergic projection from the SN to the OB, there are other possibilities. One possibility is that the intrinsic dopaminergic neurons in the OB were also ablated by the 6-OHDA SN injection. Since these OB dopaminergic neurons have an inhibitory effect on the mitral cells, loss of these dopaminergic neurons would result in excitatory effects on the mitral cells and/or ability of newly acquired odor memory. Computational modeling has demonstrated that deep short-axon dopaminergic neurons are involved in coordinating activity in the GCs and resultant activities in the mitral cells and the middle tufted cells, which together form the main output from the OB^55^. At least, we can exclude the possibility of involvement of PGCs because the number of OB PGCs was unchanged whereas the SN dopaminergic population was reduced by about 50% (Fig. 1c,d). In contrast, we could not conclude about the dorsal short axon cells, because no direct data were available. Furthermore, It is also possible that indirect pathways are involved. There are massive dopaminergic inputs from the SN and striatum to piriform cortex and the olfactory tubercle, and these areas send strong feedback projections to the OB^44,45^. Therefore, loss of dopaminergic neurons in the SN could affect the OB indirectly via these two higher olfactory centers. Thus, it is likely that the impaired olfactory-related behaviors and abnormal hyperactivity of background oscillatory LFPs and odor-evoked calcium responses in the OB are resulted from changes to both direct and indirect pathways.

In summary, after partial lesion of the dopaminergic neurons in the SN, we found deficits in olfactory-related behaviors prior to motor dysfunction. The changes in olfactory behavior were likely due to abnormal hyperactivity of the mitral cells in the OB. Further studies are needed to elucidate the details of the neural pathways underlying our observations.

## Methods

### Animals

Male C57BL/6J and Thy1-cre mice were used in this study (8–16 weeks old). The number of mice used in each experiment is reported in the Results. All mice were bred in the animal facility of Xuzhou Medical University. They were housed in a vivarium with a 12/12 light/dark cycle with lights on at 8:00 a.m. Experiments were performed during the light cycle. Food and water were available *ad libitum*.

### Injection of 6-OHDA into the SN

Mice were deeply anesthetized by an intraperitoneal injection of sodium pentobarbital (0.09 mg/g body weight, dissolved in normal saline at a concentration of 15 mg/ml) and mounted in a mouse stereotaxic frame. A dental drill was used to make a craniotomy and animals received unilateral or bilateral injections of either 1 μl 6-OHDA (H4381, Sigma; 2 mg/ml 6-OHDA dissolved in 0.9% saline containing 0.02% ascorbic acid) or equivoluminal 0.9% saline containing 0.02% ascorbic acid (sham group). Injections were made with a glass pipette and a microsyringe pump (Stoelting Quintessential Injector; Stoelting Co.) and targeted the SN (from bregma: AP, −3.0 mm; ML, −1.2 mm; DV, −4.7 mm). The glass pipette was left in place for 5 min after the injection before retraction. The SN was injected unilaterally in the apomorphine-induced rotational asymmetry experiment (Fig. 1g,h), unilaterally or bilaterally in the rotarod test (Fig. 1e,f), and bilaterally in the olfactory behavior (Fig. 2), electrophysiology (Figs 3 and 4), and fiber photometry (Fig. 5) experiments.

For LFP recording and fiber photometry experiments, a cannula (O.D = 0.48 mm, stainless steel needle, RWD, Shenzhen, China) was implanted into the right SN (see below). For these mice, 6-OHDA was injected via the canula rather than a glass pipette.

### Immunohistochemistry

Animals were anesthetized with sodium pentobarbital, and perfused with 30 ml of 0.9% saline solution followed by 30 ml of 4% (w/v) paraformaldehyde in phosphate buffer (0.1 M, pH 7.4). After removal, brains were post-fixed in 4% paraformaldehyde overnight and dehydrated gradually in 30% (w/v) sucrose solution. Brains were frozen in tissue freezing medium at −20 °C for 30 min, cut on a freezing microtome in 30 μm coronal sections and collected in ten regularly spaced series. For immunofluorescent staining, sections were incubated with rabbit anti-TH antibody (1:1000; Millipore, USA) at 4 °C overnight, and then incubated with secondary antibodies conjugated with Alexa Fluor 594 (1:500; Life, USA) for 1 h. Sections were viewed and photographed with a fluorescent microscope (Olympus IX81). To assess the number of dopaminergic neurons in the SN, TH^+^ neurons were counted in 4–6 slices from AP: −2.7 mm to AP: −4.3 mm and the average across all sections was used to represent the number of TH^+^ neurons in the SN. We compared the number of TH^+^ neurons on the 6-OHDA-lesioned side with the number of TH^+^ neurons on the contralateral side. SN TH^+^ neurons were counted in the field of view of a 10× objective. For OB TH^+^ neurons, a similar procedure was followed: TH^+^ neurons were counted in 4–10 slices from one OB and the average across all sections was used to represent the number of TH^+^ neurons in the OB. OB TH^+^ neurons were counted in the field of view of a 20× objective. Cell counting was conducted in ImageJ software.

### Locomotor activity

The rotarod task was used to evaluate locomotor activity. We used a similar protocol as previous studies with some minor modifications^32,39,56^. The rotation speed started at 5 rpm with an acceleration of 7 rpm/min and a terminal speed of no greater than 40 rpm. The task ended when the mouse fell down. During the training period, mice were trained three times a day with an interval of 1 h, for three consecutive days. After one day off, the mice were tested and the time on the rod was recorded and analyzed. Apomorphine-induced rotational asymmetry were performed as in previous studies, with some minor modifications^42,57^. Mice were tested for rotational behavior induced by apomorphine seven days after 6-OHDA injection. Apomorphine hydrochloride (0.5 mg/kg; MedChem Express) in saline was injected intraperitoneally. Mice were randomly placed in an automated rotometer, and left and right full-body turns were monitored by custom computerized activity-monitoring system. Results are expressed as the total number of rotations away from the lesioned side over a period of 30 min.

### Odor habituation/dishabituation

Mice were habituated to one odor (isoamyl acetate, diluted in mineral oil at a concentration of 0.02%) for four sequential 2-min presentations separated by 2-min inter-trial intervals, followed by a single presentation of one novel odorant (2-heptanone diluted in mineral oil at a concentration of 0.02%). The magnitude of the novelty response to the novel odor indicates the mouse’s ability to detect the odorant and discriminate it from the previous odorant (Fig. 2a1). Both odors were presented by placing 50 μl of the odorant mixture onto a cotton bud that was placed on a hole of the cage. The amount of time that the mice spent actively investigating each presented odorant was measured with a stopwatch. Active investigation was defined as directed sniffing within 1 cm of the odor source.

### Olfactory spatial memory test

This assay was performed as described previously^35,36^. Briefly, the test instrument consisted of a flat square (400 mm × 400 mm) in which four holes (36 mm in diameter) had been bored to receive plexiglass containers (36 mm inside diameter, 45 mm deep). Pentanol, decanal, dodecanal, and hexanol (Xuanguang Chemical Technology Co. Ltd., Lanzhou, China) were diluted in mineral oil (at 0.074%, 1.78%, 2.74% and 0.07%, respectively) iso as to achieve an approximate vapor pressure of 1 Pa.

Seven days after 6-OHDA or sham treatment, the mice were individually habituated to the four-hole configuration without odorants for 2 min, 3 min before the test. The test was performed over two training trials (trial 1 and trial 2) and a recall trial (trial 3) between 14:00 h and 17:00 h. In the training trials, cotton pads that had absorbed 60 µl pentanol, decanal, dodecanal, or hexanol were placed into the bottom of the plexiglass containers (in holes 1, 2, 3, and 4, respectively) and mice were allowed to explore the odorants for 6 min. The number of visits to each odorant was calculated automatically by a computer recording system that automatically records and analyzes the signals from infrared detectors around the holes. In the recall trial, the spatial locations of dodecanal and hexanol were switched and the mice were allowed to explore for another 6 min, with the number of visits to each odorant recorded once more (Fig. 2b1). The mice were returned to their home cage for 3 min between the two training trials and between the training trials and the recall trial.

In this test, hole 1 (pentanol on both training and recall trials) and hole 2 (decanal on both training and recall trials) are defined as the control odorants and hole 3 (dodecanal on the training trials, hexanol on the recall trial) and hole 4 (hexanol on the training trials, dodecanal on the recall trials) are the switched odorants. In general, normal animals visit the control and switched odorants equally during the training trials (trial 1 and 2), but significantly increase the number of visits to switched odorants in the recall trial (trials 3) because the animals perceive that the odorants in hole 3 and hole 4 have changed^35,36^.

### Buried food-pellet test

The buried food pellet test was carried out to evaluate odor detection, which can be considered to reflect the olfactory threshold. It was performed as previously described^39^. Individually housed animals were fasted for 24 h prior to the test and during the experimental period. The test was performed on the seventh day after bilateral SN injection of 6-OHDA or 0.9% saline containing 0.02% ascorbic acid. Before the test, the mouse was placed into the test cage (40 cm × 30 cm × 16 cm) for 10 min to habituate to the environment. A 200-mg food pellet was buried approximately 0.5 cm below the surface of 3-cm-deep bedding. The location of the food pellet was changed at random. In each trial, the latency to find the food pellet was defined as the time between when the mouse was placed in the cage and when the mouse discovered the food pellet and grasped it in its forepaws. The latency to dig up and eat the buried food pellet was recorded with a camera. The bedding in the test chamber was changed between trials. On the eighth day after 6-OHDA injection, a visual food pellet test was carried out to make sure that the animals did not have altered locomotor activity or motivation. This test was conducted in a similar way to the buried food-pellet test, except that the food pellet was placed on the surface of the bedding.

### Implantation of LFP electrodes and optical fibers

Mice were briefly anesthetized with sodium pentobarbital (0.09 mg/g body weight) and the depth of anesthesia was verified by toe pinch. Mice were mounted in a stereotaxic frame then the fur on the surface of the scalp from the midline of the orbits to the midpoint between the ears was removed and a hole was drilled in the skull above the right OB (from bregma: AP, 4.28 mm; ML, 1.0 mm). Two screw holes were drilled into the parietal bone.

For LFP recording, a stainless steel electrode (catalog #791000; A-M systems) was implanted into the dorsolateral region of the OB at or near the MCL (from bregma: AP, 4.28 mm; ML, −1.0 mm; DV, −2.0 mm). Two screws were inserted in the holes in the parietal bone: one screw (from bregma: AP, −1.0 mm; ML, 1.0 mm) served as the ground and reference electrode, the other screw provided additional structural support for the implant. The electrodes and screws were fixed to the bone with dental cement. A custom-made aluminum head plate was attached to the skull with stainless steel screws and dental cement.

For fiber photometry, virus (AAV-DIO-GCaMP6s, 1 μl, Brainvta, Wuhan, China) was slowly injected (50 nl/min) into the OB of Thy1-Cre mouse through a glass pipette, with a microsyringe pump (Stoelting Quintessential Injector; Stoelting Co.), at the coordinates described above. The glass pipette was left in place for an additional 10 min and then slowly withdrawn. After AAV-DIO-GCaMP6s virus injection, an optical fiber (200 μm O.D., 0.37 numerical aperture (NA); Newdoon) was placed in a ceramic ferrule and inserted towards the OB through the craniotomy (from bregma: AP, 4.28 mm; ML, −1.0 mm; DV, −2.0 mm). The ceramic ferrule was supported by a stainless steel screw and dental cement. A custom-made aluminum head plate was also attached to the skull with stainless steel screws and dental cement.

### LFP recording

LFPs were recorded from a stainless steel electrode implanted in the dorsolateral region of the OB with reference to a skull screw implanted posteriolateral to bregma. Signals were amplified (2000×, Model 3500, A-M systems), filtered at 0.1 Hz–300 Hz, and sampled at 2 kHz. LFP signals were recorded together with odor-stimulation event markers via an in-house recording system based on an NI sampling card (NI-USB-6009).

### Fiber photometry

After implantation of the optical fiber, mice were housed individually for at least 10 days for recovery from the surgery and expression of the virus. Fluorescence emission was recorded with a fiber photometry system (Thinkerbiotech, Nanjing, China) using methods similar to those described in previous studies^37^. Briefly, a laser beam from a 488-nm laser (OBIS 488LS, Coherent) was reflected by a dichroic mirror (MD498, Thorlabs), focused through a 10× objective lens (NA = 0.3; Olympus), and then coupled to an optical commutator (Doric Lenses). An optical fiber (250 mm O.D., NA = 0.37, 1.5 m long) coupled the light between the commutator and the implanted optical fiber. The laser power was adjusted to 40–60 μW at the tip of the optical fiber. GCaMP6s fluorescence emission was bandpass filtered (MF525–39, Thorlabs) and detected by a photomultiplier tube (R3896, Hamamatsu). An amplifier (C7319, Hamamatsu) was used to convert the photomultiplier tube current output to voltage, which was further filtered through a low-pass filter (35 Hz cut-off; Brownlee 440). The analog voltage signals were digitized at 500 Hz and recorded by fiber photometry software (Thinkerbiotech, Nanjing, China). With the fiber used in this study, fluorescence signals could be collected over a distance of about 0.20 mm from the fiber.

The mice used for LFP and fiber photometry recordings were head-fixed rather than freely moving because odor sampling is more reliable with head fixation. Although many studies have recorded neural activity in freely moving animals, head-fixed mice have also been used extensively in many recent studies^37^. Behavior tests have demonstrated that odor-discrimination performance appears similar under these two conditions^58^, indicating that odor-processing strategies are at least somewhat similar in the two conditions.

### Odor stimulation

Awake mice were head fixed with two horizontal bars (fixed to the head plate by two screws) and were able to maneuver on an air-supported free-floating styrofoam ball (Thinkerbiotech, Nanjing, China). Different odors were presented by an odor delivery system (Thinkerbiotech, Nanjing, China). Six odorants were used: isoamyl acetate, 2-heptanone, phenyl acetate, benzaldehyde, 2-methylbutyric acid, and heptanoic acid. The odors were dissolved in mineral oil at 1% dilution. A stream of charcoal-filtered air flowed over the oil, and the air was then diluted 1/20 by an olfactometer. The odor stimulation was synchronously controlled with the data acquisition system by a solenoid valve, which was driven by a digital to analog converter. Air or odorized air were delivered to the nose at a constant rate of 1 L/min to eliminate the effect of airflow. For each odor, 10 trials were presented with an inter-trial interval of 30 s. The duration of each odor presentation was 2 s.

### Data analysis and statistics

All the data in present study were indicated by Mean ± Standard Error. The data analyses and statistics for comparing the number of TH^+^ cells and for all behavioral experiments expect the habituation/dishabituation test are described in the Results section where these data are mentioned.

For analysis of the habituation/dishabituation data, all raw investigating values were pooled with animals and organized according to odor presentation number as previously described^33^. The raw investigating values were normalized to maximum investigating duration per mouse for each presentation (trials 1–5, Fig. 2a2,a3). The normalized data were selected for analysis because of subtle group differences in trial 1 odor investigation behavior. The maximum investigating duration was assigned a value of “1” and the lesser investigating times a proportion of 1. A one-way ANOVA was performed to assess the differences among odor-presentation trials (Fig. 2a2,a3), and the Student-Newman-Keuls method was used for further post-hoc pairwise comparisons (Fig. 2a2,a3).

A custom Matlab program was used to analyze the LFP signals. Raw data from 5 s prior to the onset of odor stimulation were used to represent spontaneous ongoing activity. A time–frequency transformation was performed on data from this 5 s window (Hanning window; FFT size, 2048; frequency resolution, 0.977 Hz), and the spectral power was calculated for each frequency resolution. As in previous studies, LFP signals were divided into different frequency bands (Fig. 3): theta (2 to 12 Hz), beta (15 to 35 Hz), and gamma (low: 36 to 65 Hz; high: 66 to 95 Hz)^11,37^. For odor-evoked beta band and high-gamma LFP responses, windows 3 s prior to and 5 s after the onset of odor stimulation were selected (Fig. 4). To obtain high resolution in both the time and frequency domains, this time course was divided into 1-s segments with 90% overlap. Time-frequency transformations was performed on these 1-s windows. The spectral power from all frequencies included within the bandwidth was averaged. For each trial, the baseline power was normalized to 1, and all the trials for each odor stimulation were averaged with respect to the normalized data. Odor-evoked activity were presented as P_1_/P_0_, where P_0_ is the power of the baseline LFP oscillation and P_1_ is the power of the odor-evoked LFP oscillation (Fig. 4).

Photometry data were exported from the photometry software as Matlab. mat files for further analysis. The data were segmented according to the onset of odor stimulation within individual trials. We derived the values of fluorescence change (ΔF/F) by calculating (F − F_0_)/F_0_, where F_0_ is the baseline fluorescence signal calculated over a 5-s control time window, which preceded the onset of odor stimulation. ΔF/F values were presented as heatmaps or average plots (Fig. 5).

### Ethical approval

All experiments were performed according to protocols approved by the Xuzhou Medical University Institutional Animal Care and Use Committee. All applicable international, national, and institutional guidelines for the care and use of animals were followed.

## Data Availability

The datasets analyzed during the current study are available from the corresponding author on reasonable request.
